# Effect of bisphosphonates on periprosthetic bone loss after total knee arthroplasty: a meta-analysis of randomized controlled trials

**DOI:** 10.1186/s12891-018-2101-z

**Published:** 2018-05-30

**Authors:** Mingmin Shi, Lei Chen, Haobo Wu, Yangxin Wang, Wei Wang, Yujie Zhang, Shigui Yan

**Affiliations:** 1 Department of Orthopaedic Surgery, Second Affiliated Hospital, School of Medicine, Zhejiang University, No.88 Jiefang Road, Hangzhou, 310009 People’s Republic of China; 2 Department of Endocrinology and Metabolism, Sir Run Run Shaw Hospital Affiliated with School of Medicine, Zhejiang University, No. 3 Qingchun Road, Hangzhou, 310009 People’s Republic of China

**Keywords:** Bisphosphonates, Total knee arthroplasty, Bone mineral density, Meta-analysis randomized controlled trial

## Abstract

**Background:**

Aseptic loosening and osteolysis are the most common indications after TKA for revision surgery. This meta-analysis which included high-quality randomized controlled trials (RCTs) aimed to analyze the effect of bisphosphonates (BPs) on maintaining periprosthetic bone mineral density (BMD) after total knee arthroplasty.

**Methods:**

PubMed, AMED, EMBASE, the Cochrane library, ISI Web of Science, and China National Knowledge Infrastructure were systematically searched, five RCTs were included and the total number of participants was 188. The weighted mean differences with 95% confidence interval were calculated to evaluate the efficacy of BPs on total BMD of knee and the BMD of different periprosthetic regions. A descriptive review was performed for BP-related adverse effects.

**Results:**

The BPs group presented significantly higher total BMD in proximal part of the tibia than the control group at 3 and 6 months (*P* < 0.05), but no significant difference at 12 months (*P* = 0.09). The BPs group presented significantly higher BMD in the distal aspect of the femur than that in the control group at 3, 6, 12 months. The BPs group presented significantly higher periprosthetic BMD than that in the control group at 3, 6 and 12 months in tibial medial and lateral metaphyseal region, and femoral anterior, central and posterior metaphyseal region (*p* < 0.05), but no significant difference for tibial diaphyseal region at 3, 6, and 12 months. None of the included studies described severe or fatal adverse effects related to BPs.

**Conclusion:**

BPs have a short-term effect on reducing periprosthetic bone loss after total knee arthroplasty. Compared with diaphyseal region, BPs are more effective on the preservation of BMD in medial lateral metaphyseal regions of proximal tibia and in anterior, central, and posterior metaphyseal region of distal femur.

## Background

Total knee arthroplasty (TKA) is a successful therapeutic option for the patients with knee osteoarthritis and rheumatoid arthritis. However, TKA changes the mechanical loads on the knee joint and causes bone mineral density and structure be adjusted to meet new mechanical demands surrounding the prosthesis and the new alignment of the lower legs [1–3]. Aseptic loosening and osteolysis are the most common indications after TKA for revision surgery [4–6]. Bone loss is mainly related to stress shielding, immobilization, and tissue damage due to surgical procedure [7, 8].

Previous studies reported a significant decrease in periprosthetic BMD after TKA [8–10].

Therefore, how to preserve the periprosthetic bone mass to improve the outcome of TKA has been an important subject [11].

Bisphosphonates (BPs) are widely used in the therapy of osteoporosis and other metabolic bone diseases. BPs are inhibitors of bone resorption which promote bone mineralization and inhibit farnesyl pyrophosphate synthase [12, 13]. Some studies revealed that bisphosphonates can decrease fracture risk and prolong survival time of implant [14, 15], and some randomized controlled trials have investigated the effect of reduce periprosthetic bone loss after total knee arthroplasty [16–20]. To confirm the effect of BPs on periprosthetic bone loss after total joint arthroplasty, we have made a previous meta-analysis [21], of which included 14 RCTs in 2012, and the result revealed that BPs could prevent bone loss after arthroplasty in medium-term follow-up. However, there were only 2 RCTs about TKA. Due to increased trend in recent investigations on effect of BPs on periprosthetic bone loss after TKA with large-scale and high quality, it is essential to update the analysis.

This meta-analysis of five high-quality RCTs aimed to analyze the effect of bisphosphonates on periprosthetic bone loss after TKA.

## Methods

### Literature search

Two independent investigators searched Electronic databases including PubMed, AMED, EMBASE, the Cochrane library, ISI Web of Science, and China National Knowledge Infrastructure from the inception dates to October 31, 2017. The search used the following keywords: (alendronate OR pamidronate OR etidronate OR zoledronate OR clodronate OR bisphosphonate) AND (arthroplasty OR knee arthroplasty OR joint prosthesis OR joint replacement OR knee replacement). To include additional eligible studies, citation lists of all the selected publications were searched by hand.

### Selection criteria

The inclusion criteria were as follows: (1) the participants underwent total knee arthroplasty, (2) the intervention was administration of bisphosphonates after total knee arthroplasty, (3) the measurements must include periprosthetic BMD, and (4) the trial design was randomized and controlled. The exclusion criteria were as follows: (1) the participants had any history of bone metabolic diseases, (2) BMD data were not available, (3) the same participants reported in a short follow-up study duplicately.

### Data extraction and outcome measures

**Fig. 1:**
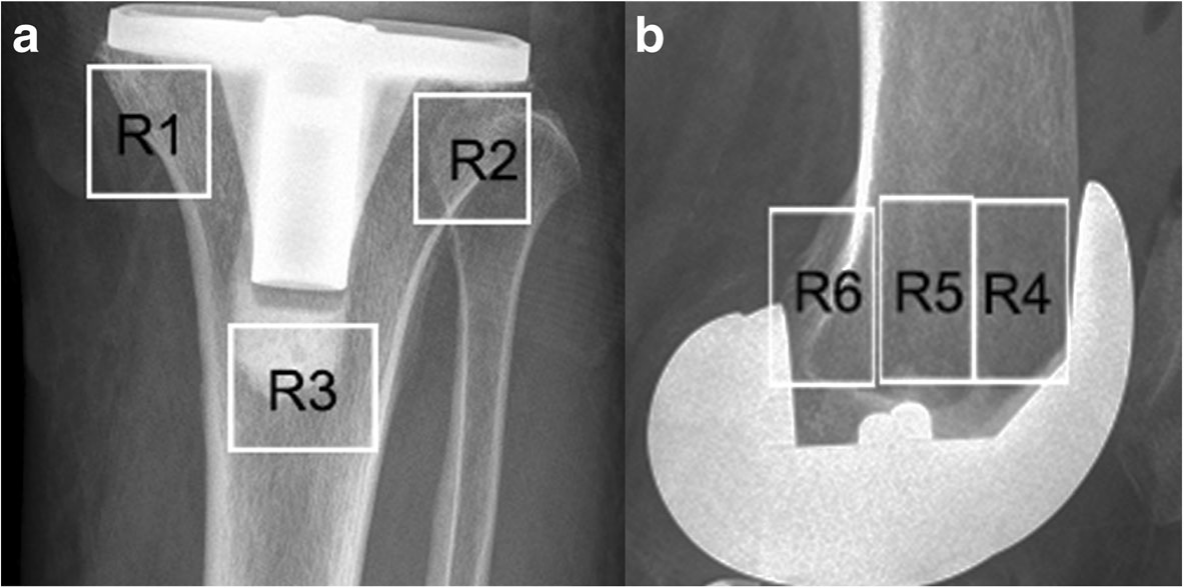
Periprosthetic ROIs of knee. **a** periprosthetic ROIs of tibia: medial metaphyseal region (R1), diaphyseal region (R2), lateral metaphyseal region (R3), (**b**) periprosthetic ROIs of femur: anterior metaphyseal region (R4), central metaphyseal region (R5), posterior metaphyseal region (R6)

For each initially screened trial, two independent investigators collected the information including name of first author, publication year, sample size, intervention, study duration, co-factors, measurements and loss-to-followup rate. If information was not described as text in the publications, we extracted it from the figures, tables, or other supplementary material. The characteristics of five finally included RCTs [16–20] were showed in Table 1. The primary outcome measurement was total periprosthetic BMD of knee. And the secondary measurement was the BMD of different periprosthetic regions of interest (ROIs), including the tibial regions and the femoral regions (Fig. 1). The tibial regions including medial metaphyseal region (R1), lateral metaphyseal region (R2), and diaphyseal region (R3). The femoral regions including anterior metaphyseal region (R4), central metaphyseal region (R5), and posterior region (R6). Because BMD levels are affected by gender, weight and general bone loss, the results were presented as a percentage of the BMD changing from the baseline. The percentage of BMD changing was used rather than the absolute numerical value to decrease the bias of different baseline. The percentage of BMD changing was calculated as follows: 100*(BMD_n_-BMD_0_)/BMD_0_. Here BMD_0_ refers to the baseline BMD value, and BMD_n_ stands for the postoperative BMD at certain follow-up time point. Sensitivity analysis was performed for the effect size by omitting the studies for which data were imputed.

**Table 1:** Characteristics of the included studies

Study	Age (years), I/C	Sample Size, I/C	Intervention	Follow-up (month)	Outcome Measures
Soininvaara 2002	83.5 ± 19.9/ 79.7 ± 8.7	19 (8/11)	10 mg/day oral alendronate+ 500 mg/day calcium carbonate for 12 months vs. 500 mg/day calcium carbonate	12 months	Periprosthetic BMD: proximal femur
Han 2003	63.6 ± 4.1/ 65.2 ± 5.6	72 (36/36)	10 mg/day oral alendronate for 6 months vs. no placebo	12 months	Periprosthetic BMD: distal part of femur and proximal aspect of tibia
Wang 2006	69.8 ± 5.9/ 69.7 ± 6.7	60 (30/30)	10 mg/day oral alendronate for 6 months vs. no placebo	3 years	Periprosthetic BMD:distal part of femur and proximal aspect of tibia
Abu-Rajab 2009	68 ± 2.2/ 72 ± 8.1	11 (5/6)	70 mg/week oral alendronate for 6 months vs. a placebo	2 years	Periprosthetic BMD: distal part of femur and proximal aspect of tibia
Jaroma 2015	66 ± 7.0/ 68 ± 8.2	26 (14/12)	10 mg/day oral alendronate+ 500 mg/day calcium carbonate for 12 months vs. 500 mg/day calcium carbonate	7 years	Periprosthetic BMD: distal part of femur and proximal aspect of tibia

*I/C* intervention/control groups, *BMD* bone mineral density

### Methodological quality assessment

The methodologic quality of included trials were assessed the by two investigators independently with the Cochrane Collaboration tool for risk of bias, in which assessing factors included randomization, allocation concealment, and blinding etc. The weighted kappa for the agreement on the assessment of quality between all reviewers was 0.89 [95% confidence interval (CI), 0.80–0.99]. The criteria of the Grading of Recommendations Assessment, Development and Evaluation (GRADE) were used to assess the quality of evidence [22].

### Statistical analysis

For data reported as continuous variables, means and standard deviations were extracted. All extracted data were input and analyzed in Review Manager 5.3.5 version (Cochrane Collaboration, London, England). Chi-square test and I^2^ was used to assess heterogeneity the [23]. When there was no statistical heterogeneity (*P* < 0.10, or I^2^ < 50%), the fixed-effect (FE) model was used; otherwise (*P* > 0.10, or I^2^ > 50%), a random-effect (RE) model was chosen [24].

Sensitivity were evaluated by omitting some trials to assess whether specified factors (small sample size, randomization, intention-to-treat (ITT) analysis *etc*) could affect the overall result of analysis. The *P* value of heterogeneity less than 0.05 was considered as significant differences. The analyses of sensitive could not be performed when the number of trials was less than three in comparison.

## Results

### Trials selection

**Fig. 2:**
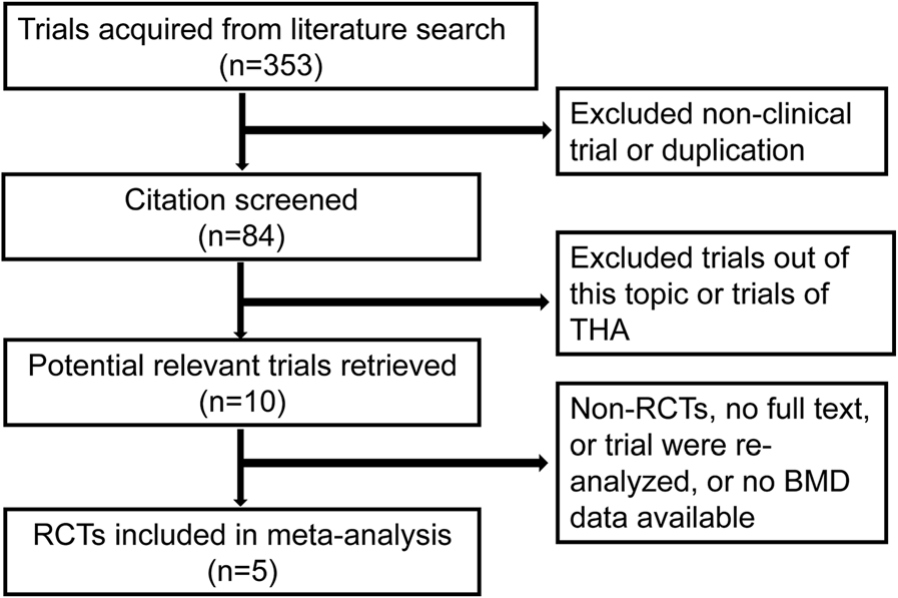
The flowchart of the selection of 5 randomized controlled trials included in the presented meta-analysis

A flow diagram illustrating the study identification is shown in Fig. 2. There were 353 relevant trials selected by initially search, of which 269 trials were excluded because they were duplicated or non-clinical trial. Of the 84 remaining articles, only 10 studies were on the main topic. Among the 10 studies, one trial [25] was excluded because there were longer follow-up and re-analyzed studies reported the same participants. But the data from these shorter-term follow-up trials were considered to be used when they were analyzed in other later trials. Two excluded trials had no available BMD data [26, 27]. Two trials were excluded because they were non-RCTs [14, 28]. Finally, 5 RCTs involving 188 participants were included in our meta-analysis (Fig. 1). The weighted kappa for agreement on eligibility between reviewers was 0.88 (95% CI, 0.80–0.96).

The characteristics of the included trials were summarized in Table 1. The BPs used in all the five trials were alendronate.

### Methodological quality

**Fig. 3:**
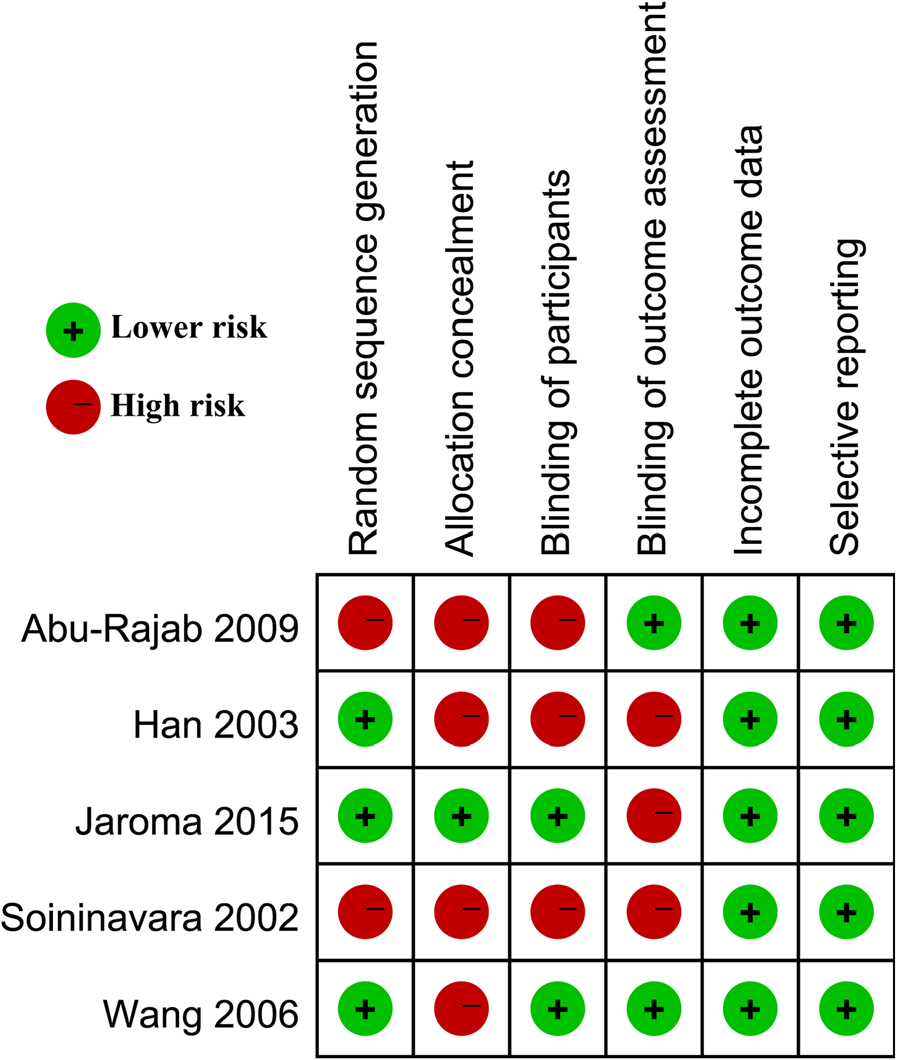
Risk of bias summary: review authors’ judgements about each risk-of-bias item for each included study

A 6-item scale for assessing methodological quality was used (Fig. 3). All the 5 trials were RCTs. Three studies described adequate randomization, only one study demonstrated sufficient allocation concealment, two studies described the blinding of outcome assessment and two studies described the blinding of participants. All the five studies retained complete outcome data, avoided selective reporting, and seemed to be free of other potential sources of bias. The investigators achieved good agreement in evaluating the methodological quality (0.86, 95% CI: 0.82–0.90).

### Preservation of total periprosthetic BMD

**Fig. 4:**
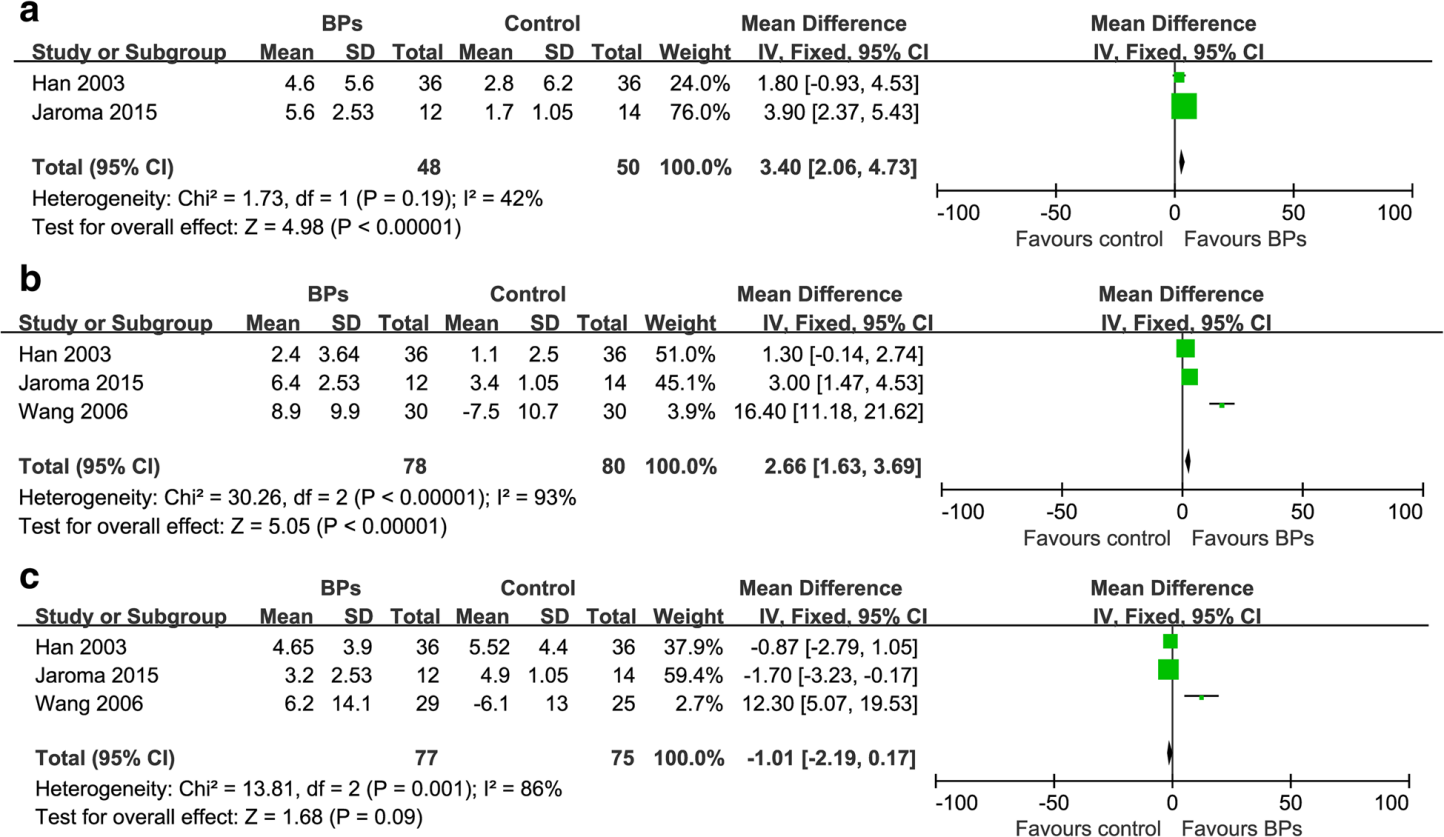
Forest plots for the effect of BPs on total periprosthetic BMD in proximal tibia at 3 months (**a**), 6 months (**b**) and 12 months (**c**)

**Fig. 5:**
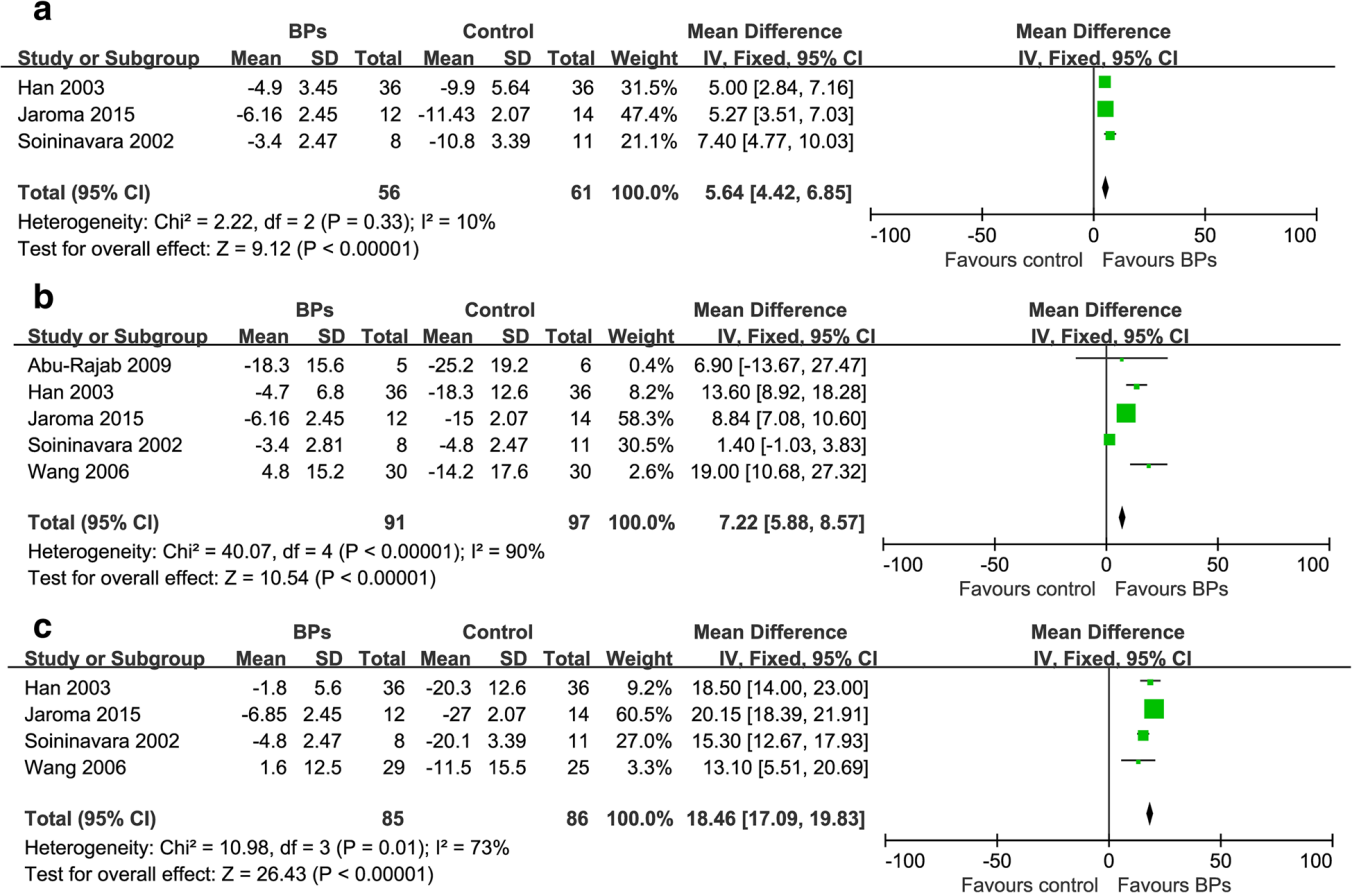
Forest plots for the effect of BPs on total periprosthetic BMD in distal femur at 3 months (**a**), 6 months (**b**) and 12 months (**c**)

It was illustrated that the BPs group presented significantly higher total BMD in proximal part of the tibia than the control group at 3 and 6 months respectively [(2 trials, WMD: 3.40, 95% CI: 2.06–4.73, *p* < 0.05); (3 trials, WMD: 2.66, 95% CI:1.63–3.69, *p* < 0.05)] (Fig. 4a and b). There was no significant difference of total BMD in proximal part of the tibia between the BPs group and the control group at 12 months (3 trials, WMD: -1.01, 95% CI: -2.19-0.17, *p* = 0.09) (Fig. 4c). The BPs group presented significantly higher BMD in the distal aspect of the femur than that in the control group at 3, 6, 12 months [(3 trials, WMD: 5.64, 95% CI: 4.42–6.85, *p* < 0.05); (5 trials, WMD: 7.22, 95% CI: 5.88–8.57, *p* < 0.05); (4 trials, WMD: 18.46, 95% CI:17.09–19.83, *p* < 0.05)] (Fig. 5).

### Preservation of BMD in different knee regions

It was illustrated that the BPs group presented significantly higher periprosthetic BMD than that in the control group at 3, 6 and 12 months in R1, R2, R4, R5 and R6 (*p* < 0.05) (Table 2). For R3, there was no significant difference of periprosthetic BMD between the BPs group and the control group at 3, 6, and 12 months (*p* < 0.05).

**Table 2:** Meta-analyses of BMD of the different regions of knee at different time points

ROI		3 months	6 months	12 months
		WMD (95%CI)	WMD (95%CI)	WMD (95%CI)
Tibia	1	4.90 (3.53, 6.26)	6.63 (5.34, 7.93)	4.22 (2.92, 5.51)
		*P* < 0.05	*P* < 0.05	*P* < 0.05
	2	2.61 (1.32, 3.91)	5.86 (4.57, 7.16)	8.57 (7.28, 9.87)
		*P* < 0.05	*P* < 0.05	*P* < 0.05
	3	1.10 (−0.19, 2.40)	0.45 (− 0.84, 1.75)	0.36 (0.94, 1.65)
		*P* = 0.18	*P* = 0.39	*P* = 0.44
Femur	4	4.10 (2.74, 5.46)	4.50 (3.13, 5.86)	5.70 (4.34, 7.06)
		*P* < 0.05	*P* < 0.05	*P* < 0.05
	5	6.90 (5.54, 8.27)	6.50 (5.14, 7.87)	7.02 (5.66, 8.39)
		*P* < 0.05	*P* < 0.05	*P* < 0.05
	6	4.34 (2.97, 5.70)	4.32 (2.96, 5.68)	5.25 (3.89, 6.62)
		*P* < 0.05	*P* < 0.05	*P* < 0.05

ROI: region of interest, WMD: weighted mean differences

A positive value of WMD means it favors experimental, and negative value means favoring control

### Heterogeneity and sensitivity analysis

The BMD in proximal part of the tibia and the distal aspect of the femur at different follow-up were statistically heterogeneous at 6 (tibia: χ^2^ = 30.26, *p* < 0.00001, I^2^ = 93%; femur: χ^2^ = 40.07, *p* < 0.00001, I^2^ = 90%) and 12 months respectively (tibia: χ^2^ = 13.81, *p* < 0.00001, I^2^ = 86%; femur: χ^2^ = 10.98, *p* < 0.00001, I^2^ = 73%) but not at 3 months (Fig. 4 and Fig. 5). The heterogeneity could not be minimized by omitting any trial. The overall effect was not significantly altered by omitting trials without the ITT analysis, those with small sample size (less than 20), or those funded by companies.

Strength of evidence.

### Adverse reaction

No serious adverse effect was reported related to BPs in the 5 trials. The mostly reported adverse effect was digestive discomfort in 3 trials: 1 of 8 by Soininvaara [16]; 5 patients by Wang [18]; 2 patients by Jaroma [20]. There was no severe side effect on renal, hepatic, or heart function.

## Discussion

High BMD supports bone-implant fixation, and there have been several attempts to improve the quality of the primary arthroplasty and to reduce the incidence of failures caused by loss of BMD [29, 30]. A large population-based parallel-cohorts study [31] found that bisphosphonates could decrease the risk of periprosthetic fractures after THA. A larger retrospective cohort study on participants with primary total hip/knee arthroplasty showed that oral bisphosphonates reduced risk of revision surgery by 59% [32]. The present meta-analysis strengthened the evidence of BPs reducing periprosthetic bone loss.

Our previous meta-analyses based on 14 RCTs [21] found that BPs significantly preserved total periprosthetic BMD up to 10 years after joint arthroplasty. However, most included trials in that meta-analyses were in regards to THA, and there was only 2 RCTs about the TKA. More significant efficacy in proximal tibia was found at 3 and 6 months after arthroplasty in the BPs group compared with the control group. However, this difference was not significant at 12 months after arthroplasty. The reason that later stages after surgery have respectively lower efficacy may be the active bone resorption caused by the early iatrogenic damage and the late stress shielding induced osteolysis [33]. Meanwhile, more significant efficacy in distal femur was found at 3, 6 and 12 months after arthroplasty in the BPs group compared with the control group. Moreover, the secondary finding is that different femoral region has different response to bisphosphonates. In the proximal tibial region R1 and R2, BPs group presented significantly higher periprosthetic BMD than that control group up to 12 months after arthroplasty, and in the proximal tibial region R3, the difference between BPs group and control group was not significantly at 3, 6 and 12 months after arthroplasty. The possible interpretation is that the medial and lateral metaphyseal region have more stress shielding than diaphyseal region [34]. In the distal femoral region R1, R2 and R3, BPs group presented significantly higher periprosthetic BMD than that control group at 3, 6 and 12 months after surgery. The possible interpretation is that these three regions have similar mechanical environment which provides similar stress shielding [35]. The present study showed there was no serious adverse effect related to BPs. It was reported a higher risk of periprosthetic fractures was found in TKA patients who used BPs, but the numbers were very small [36]. As there was no atypical femur fracture observed in this meta-analysis, there are likely to be a variety of factors involved in peri-prosthetic fracture, such as femoral geometry, prolonged duration of BP use, smoking, and activity level.

The strengths of our meta-analysis include the most included trials and largest sample size investigating the effect of BPs treatment following TKA. According to the GRADE system for evidence quality, all the included trials in the present meta-analysis began as high-quality or moderate-quality evidence, which was downgraded by five categories of limitations (Table 3). Inadequate blinding and substantial loss follow-up in some trials may raise risk of bias. Inconsistent reporting of outcomes and significant heterogeneity might reduce the quality. The number of included patients less than 150 is considered to be small and may cause imprecision and effect size more than 0.05 is considered to be large and strengthen the evidence.

**Table 3:** GRADE evidence profile of RCTs for effect of BPs on periprosthetic bone loss after TKA

	Summary of findings			Quality assessment					
	Time points	n (treated/control)	WMD (95%CI, g/cm^2^)	Limitations	Inconsistency	Indirectness	Imprecision	Others	Quality
Tibia	3 months	2 (48/50)	0.07 (0.04–0.10)	No serious^a^	No serious^b^	No serious	Serious^c^	Strong association^d^	Moderate
	6 months	3 (78/80)	0.12 (0.10–0.15)	No serious	No serious	No serious	No serious	Strong association^d^	High
	12 months	3 (77/75)	0.12 (0.10–0.15)	No serious	Serious	No serious	No serious	None	Moderate
Femur	3 months	3 (56/61)	0.07 (0.04–0.10)	No serious	No serious	No serious	Serious	Strong association	Moderate
	6 months	5 (91/97)	0.12 (0.10–0.15)	No serious	Serious	No serious	No serious	Strong association	Moderate
	12 months	4 (85/86)	0.12 (0.10–0.15)	No serious	Serious	No serious	No serious	Strong association	Moderate

^a^The inadequate blinding and substantial loss follow-up in some trials may raise risk of bias

^b^Inconsistent report of outcomes and significant heterogeneity existed across the trials

^c^The number of included patients less than 150 is considered to be small and may cause imprecision

^d^Effect size more than 0.05 is considered to be large and strengthen the evidence

The present meta-analysis has several limitations. Firstly, the limited number of trials and small sample size in some trials might reduce the precision of the pooled estimates. Secondly, the inclusion criteria and baseline characteristics of the included trials were heterogeneous, including the primary diseases, gender, ages of patients and the type of prosthesis, which would lead to bias. Thirdly, trials included in this meta-analysis only used alendronate. Finally, the presented study analyzed the short-term effect of BPs on periprosthetic bone loss after TKA, and the long-term effect remained unknown and required more clinical studies.

## Conclusion

In the present meta-analysis of randomized clinical trials, BPs have a short-term effect on the preservation of periprosthetic BMD after total knee arthroplasty. Compared with diaphyseal region, BPs are more effective on the preservation of BMD in medial lateral metaphyseal regions of proximal tibia and in anterior, central, and posterior metaphyseal region of distal femur.

## Abbreviations

BMD: Bone mineral density
BPs: Bisphosphonates
CI: Confidence interval
FE: Fixed-effect
GRADE: Grading of Recommendations Assessment, Development and Evaluation
ITT: Intention-to-treat
RCTs: Randomized controlled trials
RE: Random-effect
ROIs: Regions of interest
TKA: Total knee arthroplasty
WMD: Weighted mean difference
